# Supplementary material for: Molecular characterization and antibiotic susceptibility of Shiga toxin- producing Escherichia coli (STEC) isolated from raw milk of dairy bovines in Khyber Pakhtunkhwa, Pakistan
Source: PLoS One. 2024 Sep 3;19(9):e0307830. doi: 10.1371/journal.pone.0307830 (PMC11371208; doi:10.1371/journal.pone.0307830)
Supplement: Attachment — Submitted filename: Response to Reviewers..docx [file pone.0307830.s001.docx]

Thank you for sending reviewers’ comments on our manuscript on,

**ONE-D-23-43641**

**Molecular Characterization and Antibiotic Susceptibility of Shiga Toxin- Producing Escherichia Coli (STEC) Isolated from Raw Milk of Dairy Bovines in Khyber Pakhtunkhwa, Pakistan**

**PLOS ONE “**

We have revised our manuscript according to these comments. Answers to each of the comments are given below. I hope that this revised manuscript is now found suitable for publication,

**Reviewer 1 Comments**

1. Please ensure that your manuscript meets PLOS ONE's style requirements, including those for file naming.

**Answer.** The manuscript has been completely revised in a proper PLOS one Style. The text presentation has been refined, inconsistencies have been eliminated, unity has been improved, and overall readability has been enhanced.

2. Please provide a complete Data Availability Statement in the submission form,

**Answer**: Data availability statement submission form.

3. PLOS ONE now requires that authors provide the original uncropped and unadjusted images underlying all blot or gel results reported in a submission’s figures

**Answer:** original uncroped gel results figures has been attached.

4. PLOS requires an ORCID ID for the corresponding

Answer: ORCID iD is enlisted (0000-0002-96225217)

5. Please amend the manuscript submission data (via Edit Submission) to include author "Muhammad Hasnain Riaz".

**Answer**: author name is included as per your suggestion.

6. We note that Figure 4 in your submission contain map images which may be copyrighted. All PLOS content is published under the Creative Commons Attribution License (CC BY 4.0), which means that the manuscript, images, and Supporting Information files will be freely available online

Answer: Figure 4 has been changed and new non map images attached..

5. Review Comments to the Author

Please use the space provided to explain your answers to the questions above. You may also include additional comments for the author, including concerns about dual publication, research ethics, or publication ethics. (Please upload your review as an attachment if it exceeds 20,000 characters)

**Answer:** needful done as per your suggestion.

Reviewer #1: Line 33 Carried the virulence genes bla CTXM and bla TEM - These are antibiotic resistance genes

**Answer**: needful done as per your suggestion.
Line 36 - 37 Multi Locus Sequence Typing (MLST) was performed on twelve (12) - why only 12? why not all 40?

**Answe**r: MLST was performed on twelve isolates, for genome analysis. While the funding issue for genomic analysis.
Line 55, 58, 61, 64, 70 - , as documented by (delete)

**Answer:** as per suggestion needful done.
Line 63 - it's important to note
Line 78 - prevalence of STEC in Pakistan has been reported to vary significantly in various food products..

**Answer:** needful done.
Line 79 - Antimicrobial resistance is seriously challenging the global health (rephrase this sentence)

**Answer**: rephrase has been done the sentences,
Line 82 - Enterobacteriaceae (remove italics) rephrase sentence. It's not making sense.
Line 90 - as documented (FAO 2007) Use more recent refs.
Line 91 -Therefore forty isolated of these bacteria from raw milk from... (Rephrase this sentence)
Line 109 - United States of America Materials and Methods
Line 119 - Indole
Line 120 - Voges Proskauer (VP)
Line 130 - Genomic DNA was extracted using the boiling method (17)
Line 146 - to perform an in silico molecular characterization of the sequenced strains. Please indicate which strains were sequenced and on which platform. Also, be clear on the bioinformatics tools that where used for analysis.
Line 163-164 - Unnecessary statement. Delete.
Line 167 - Kirby Bauer CLSI 2013. Why would you use guidelines from over 10 years ago? Please use more recent guidelines.
Line 172 - Antibiogram of the ESBL positive E. coli isolates was performed using antimicrobials (rephrase sentence)
Line 173, 178/179 - Put antibiotic disc concentrations
Line 184 - In Fig. 4
Line 184 - The study identified an E. coli prevalence rate of 40.5% among these samples are described in Table 1, and Fig:1 (rephrase this sentence)
Fig 2 - Very poor fig which is poorly annotated.
Fig 3 - poorly annotated figure. Say more about the presented isolates
Fig 4 - Poor figure. Put a key.
Table 2 - rfb gene mentioned for the first time in this table. The table needs fixing. PCR is a molecular technique, reagents are mixed at microlitre quantities not milliliters.
Table 3 - frequency of positive genes not adding up to 40.
Table 4 - Some antibiotic spellings are wrong e.g Sulphamathoxole, Oxytetracyclin, Ceftriexone. Put antibiotic concentrations. Why use Amoxy & AMC?
Line 257 - Define multi-drug resistance.
Line 267 - However, despite this constraint, the discovery of both bla TM and bla CTXM genes parallel is remarkable (rephrase the sentence). Why is the presence of these genes remarkable?

**Answer:** Your comments have been duly addressed and highlighted, and appropriately relocated to the material methods and results chapters after thorough revision.

**Reviewer 2 Comments**

 I regret to disappoint authors, but due to numerous methodological errors and ambiguities in the experiment described in the manuscript, I do not recommend this manuscript for publication.
- the authors' goal was to isolate and identify E. coli strains producing shiga toxin, i.e. STEC, from raw milk, because these are strains that may be responsible for various serious infections in humans.
I suggest changing the style in which the introduction was written a bit and removing sentence endings such as: as observed by, as highlighted, as discussed by, according to. They are unnecessary and make understanding difficult. Moreover, authors in such cases should refer to the names of the cited authors, not reference numbers
Lines: 99-102: goal description should be simplified; the sentence is too long and incomprehensible

**Answer:** Both chapters have been revised and repetition has been removed, and strong links among sentences have been developed as per your comments.

Line 112: the authors performed selective pre-isolation in the presence of 0.1 mg/ml cefotaxime. According to the CLSI standard, the screening confirmatory test for ESBLs in E. coli uses a 100x lower concentration of cefotaxime. Therefore, with such selective multiplication, most E. coli strains will not grow - a serious methodological error that excludes further results obtained by the authors.
Line 146: authors write about molecular characterization based on sequencing. No description of the sequencing procedure (methodology, which strains?)
Line 114: how to check turbidity in a cloudy, even diluted, milk sample?
Lines 157-163: no description of MLST procedure (only genes listed)
Antimicrobial susceptibility profiles:
The procedure for assessing drug susceptibility using the disc diffusion method is a standardized procedure, similar to the screening and confirmatory test for ESBL (the authors described a completely different methodology).
- antimicrobials used only in veterinary medicine were used, for which there are no criteria in the M100S standard. For some antibiotics used in human medicine there are no criteria in this standard either. So what criteria were used to assess drug susceptibility?
Lines 211-212: whether classical serotyping or molecular methods were used to determine serotypes and serogroups? The authors mix the two versions in “materials and methods” vs “results”
Lines217-221: please provide the specific STs obtained.
Due to the presented inconsistencies, the discussion and conclusion do not seem credible.

**Answer.** Changes have been made and followed the suggestion.
Minor comments:
Please use susceptible instead of sensitive and antimicrobials instead of antibiotics.
Lines 93-95: I suggest deleting these sentences, the authors did not perform WGS
Lines 152-155: I suggest deleting it

Answer. Changes has been made according to suggestion
