# Supplementary material for: Molecular characterization and antibiotic susceptibility of Shiga toxin- producing Escherichia coli (STEC) isolated from raw milk of dairy bovines in Khyber Pakhtunkhwa, Pakistan
Source: PLoS One. 2024 Sep 3;19(9):e0307830. doi: 10.1371/journal.pone.0307830 (PMC11371208; doi:10.1371/journal.pone.0307830)
Supplement: Attachment — Submitted filename: Rebuttal Response to reviewers.docx [file pone.0307830.s002.docx]

To

Sherin Reda Rouby, PhD
Academic Editor
PLOS ONE

Subject:	**PONE-D-23-43641R1”Molecular Characterization and Antibiotic Susceptibility of Shiga Toxin- Producing Escherichia Coli (STEC) Isolated from Raw Milk of Dairy Bovines in Khyber Pakhtunkhwa, Pakistan PLOS ONE**

Dear Dr. Sherin,

Thank you for reviewing the manuscript and for providing clear instructions on the revisions needed. I appreciate your time and guidance.

A strenuous effort has been made to address all the comments raised by the reviewers to improve the overall quality of manuscript to be published in PLOS ONE journal.

| ***Molecular Characterization and Antibiotic Susceptibility of Shiga Toxin- Producing Escherichia Coli (STEC) Isolated from Raw Milk of Dairy Bovines in Khyber Pakhtunkhwa, Pakistan PLOS ONE*** **manuscript on, ONE-D-23-43641**  Thank you for sending reviewers’ comments on our manuscript.  We have revised our manuscript according to these comments. Answers to each of the comments are given below. I hope that this revised manuscript is now found suitable for publication. | | |
| --- | --- | --- |
| **S. #** | **Comments** | **Reply** |
|  | Comments to the author |  |
|  | 1. If the authors have adequately addressed your comments raised in a previous round of review and you feel that this manuscript is now acceptable for publication, you may indicate that here to bypass the “Comments to the Author” section, enter your conflict of interest statement in the “Confidential to Editor” section, and submit your "Accept" recommendation. |  |
|  | Reviewer 1 (No response) | Not Applicable (N/A) |
|  | 2. Is the manuscript technically sound, and do the data support the conclusions? The manuscript must describe a technically sound piece of scientific research with data that supports the conclusions. Experiments must have been conducted rigorously, with appropriate controls, replication, and sample sizes. The conclusions must be drawn appropriately based on the data presented. |  |
|  | Reviewer 1 Partly | Thank you for the critical review and suggestions, which have been incorporated to enhance the quality of the manuscript to meet the esteemed journal's criteria. The experiments were conducted in various institutes as required, ensuring that all references are included in the manuscript. Adequate sample sizes and appropriate control measures were applied, and the results support the conclusions, making a significant contribution to the research database for further study. The revised version hopefully fulfills all criteria. |
|  | 3. Has the statistical analysis been performed appropriately and rigorously? |  |
|  | Reviewer #1: N/A | N/A |
|  | 4. Have the authors made all data underlying the findings in their manuscript fully available? The [PLOS Data policy](http://www.plosone.org/static/policies.action#sharing) requires authors to make all data underlying the findings described in their manuscript fully available without restriction, with rare exception (please refer to the Data Availability Statement in the manuscript PDF file). The data should be provided as part of the manuscript or its supporting information, or deposited to a public repository. For example, in addition to summary statistics, the data points behind means, medians and variance measures should be available. If there are restrictions on publicly sharing data—e.g. participant privacy or use of data from a third party—those must be specified. |  |
|  | Reviewer #1: No | Thank you for your review and suggestion. We have thoroughly revised the manuscript to adhere to the proper PLOS One style. The text has been refined for better presentation, inconsistencies have been addressed, unity has been improved, and overall readability has been enhanced. Additionally, the Data Availability Statement is now fully presented in the supplementary file, ensuring that all information is readily accessible without restrictions. |
|  | 5. Is the manuscript presented in an intelligible fashion and written in standard English? PLOS ONE does not copyedit accepted manuscripts, so the language in submitted articles must be clear, correct, and unambiguous. Any typographical or grammatical errors should be corrected at revision, so please note any specific errors here. |  |
|  | Reviewer #1: No | Thank you for your critical review and comment. The revised manuscript has been carefully crafted to remain to the standard English format required by the journal. We have addressed language clarity and corrected grammatical errors during revision. We are confident that the manuscript now meets the publication criteria of the journal. |
|  | 6. Review Comments to the Author Please use the space provided to explain your answers to the questions above. You may also include additional comments for the author, including concerns about dual publication, research ethics, or publication ethics. (Please upload your review as an attachment if it exceeds 20,000 characters) | Thank you for your critical review and comment.  Regarding the research study, we assure you of its genuineness and the dual publication of its data. All ethical considerations have been meticulously addressed in accordance with the journal's criteria. Additionally, we have fulfilled all necessary requirements. |
|  | Reviewer #1: Check paper for gramma especially abstract, but the whole paper needs to be checked and corrected. In the initial manuscript there was mention of the ESBL genes bla CTX-M and bla-TEM but they’ve been removed in this revised version. Why? Was the detection of antibiotic resistance genes not done at all? There was also mention of extracting DNA using the boiling method which has also been removed. Does this mean extraction was only done using the DNA kit mentioned in the revised version of the manuscript? Always make an effort to address all comments by the reviewers as they are meant to improve the quality of your work. Wrong spellings e.g antibiotics, poorly annotated diagrams and weak grammar make the work less appealing. There’s need to improve on the technical aspects of the manuscript. The presentation of results needs improvement. | Thank you very much for the comments made in the manuscripts. I really appreciate your critical feedback.  The ESBL genes blaCTX-M and blaTEM were initially mentioned, but due to lack of supportive data, we have removed them from the manuscript. Antibiotic susceptibility testing was performed instead of genotyping the resistant genes.  For isolation of DNA extraction for normal PCR is mostly done through the boiling process. However, for MLST, genomic DNA extraction was carried out using extraction kits. And DNA kit mentioned in the revised version of the manuscript.  We acknowledge the importance of addressing all reviewer comments to enhance the quality of our work. We addressed and corrected the spelling errors, tried to improve diagram annotations, and strengthen the grammar to make the manuscript more appealing. Additionally, we focused on enhancing the technical aspects and improving the presentation of our results. |
|  | Line 28 Rephrase sentence. A total of 800 bovine raw milk samples from milk shops (500), ... Line 30 write CT-SMA in full when writing for the first time. Line 30 the positibve isolates were subjected to Line 31 in silico Line 32 rephrase sentence. 158 isolates were checked why are the percentages out of 800? Line 34 say something about the listed serogroups Line 37 ceftriaxone (correct spelling). Put percentages in brackets. Put a full stop after penicillin (44.5%). Line 41 list the identified sequence types Line 43 ...had the potential ability to transfer antibiotic resistance and virulence genes. This assertion is based on what exactly? Line 132 the 0.5 MacFarland’s standard not 0.05 Line 138 indole, Voges Proskauer (VP) Line 140 justify why you used the concentration of antibiotic you used. 0.1 gm of cefotaxime (CT-SMAC). Was it based on previous studies or was developed in this study. Lines 144, 145 A subset of 40 samples matching to the same sampling years as the dairy bovine raw milk isolates was selected and subjected to sequencing. What do you mean by same sampling years?? Wasn’t selection based on the isolates that were positive in the STEC mPCR? Lines 156 – 158 17µl Nuclease free water + 3µl DNA + 2.5µl PCR buffer + 1µl primers + 2µl dNTPs + 0.5µl Taq = 26µl in total. Please cross check and correct. Which enzyme (manufacturer, city) was used in the PCR? Line 168 Put antibiotic disc concentrations Line 171 and the zones... complete the sentence.  172 The CLSI document is an official document that can be cited independently. This must be done here.  Line 174 What was the selection criterion used to select the 12 isolates. This should be made clear.  Line 178 You need to put the web addresses of the tools you used for analysis e.g PATRIC (http://www.patricbrc.org). However PATRIC is now housed under BV-BRC (https://www.bv-brc.org/). Specify the specific tool/s you used for assembly, did you use SPAdes? Please provide all more detail on the bioinformatics analysis so that others may follow what you did.  Line 199 stx1, stx2, eae, ehxA. genes in table 2 and figure 2. Please correct this sentence Line 200 and street vendors were and... Please correct this sentence Line 202 100% (40/800) – this is not 100% Line 204 All raw milk samples showed the presence of ... All means 100%. You can’t say all and then put a % which lower than 100%. Please correct this. Lines 210 & 211 are the percentages susceptibility or resistance?Line 212 ceftriaxone (16%) and were highly resistant to amoxicillin (100%)  Line 214 You’ve already mentioned the high resistance to amoxicillin or you meant to write amoxicillin and clavulanic acid (AMC)  Figure 3 – Poorly annotated. Lane 3 has pcr amplicons yet its said to be the negative control. Lanes 9 and 10 have the DNA ladder and can not be test samples. The figure casts doubts over the mPCR results presented in text. Figure 4. Say something about the isolates in the legend. Multi locus sequence types typically are denoted by ST not SF. Table 1 - rfb gene mentioned for the first time in this table  Table 5 - Some antibiotic spellings are wrong e.g Sulphamathoxole, Oxytetracyclin, Ceftriexone. | Thank you very much for the comment.  The sentence has been rephrased. Please see line No. 28- 41 edited in red colored texts for ready reference please.  Line 43 .The capacity to potentially transfer antibiotic resistance and virulence genes, examined through comprehensive genome sequencing and analysis.  The sentence from line 132 has been corrected in lines 201-203 and the changes are marked in red for your quick reference.  The sentence from line 138 has been revised and corrected in lines 168-169. The changes are marked in red for your convenience."  The sentences from lines 144-145 have been changed, corrected, and highlighted in red. Please refer to lines 199-203 for the revised text.  The lines 156-158 have been revised. The concentration of PCR buffer and primers used has been corrected and highlighted in red text on lines 210-213 for easy reference.  The antibiotic disc concentrations have been added to line 168 of the manuscript. Please refer to lines 230-232 for the revised text.  Line 171 has been revised with corrections, and the description of the zones of inhibition is now included in the sentence. Please refer to lines 204-214 for details. The CLSI document references for line 172 have been documented. Please see line 234 for details.  Line 174 has been changed, and the criteria for antibiotics include a total of 12 types available in the market. Please refer to lines 231-232 for the text highlighted in red.  Bottom of Form  Line 178 has been updated with corrections and includes detailed information on next-generation sequencing (NGS) technology using the Illumina MiSeq platform by Illumina Inc., conducted at the University of Minnesota Genomic Center in the United States. Raw sequence data was processed using FASTAQ software, followed by de novo assembly using the PATRIC web tool and annotation with Prokka (version 1.13). Please refer to lines 220-221 for more information.  Line 199 has been revised with corrections. Please refer to lines 240-241 for the text highlighted in red for your reference.  The sentence at line 200 concerning street vendors has been corrected. Please refer to lines 242-244 for highlighted text for your convenience.  The lines 202 to 204 have been changed, sentences have been revised and corrected, and please refer to lines 246-252 for highlighted text for ready reference.  Lines 210 & 211 have been revised and edited. Please refer to lines 259-270 for ready reference  Line 214 have been revised and changed amoxicillin and Clavulanic acid (AMC) see line 266 for ready reference.  Figure 3 has been updated with properly marked of PCR amplicons. Please see figure no 3.  Figure 4: MLST designated the sample sequence type as ST, but the samples were marked as SF during Samples library preparation and laboratory identification for whole genome sequencing.  Table 1 includes the rfb gene, which was part of our research for identifying STEC O157 strains, but no findings were made. Therefore, its inclusion was necessary.  Table 5 The spelling of antibiotics has been corrected. Please refer to it for references, |
|  | 7. PLOS authors have the option to publish the peer review history of their article ([what does this mean?](https://journals.plos.org/plosone/s/editorial-and-peer-review-process#loc-peer-review-history)). If published, this will include your full peer review and any attached files. If you choose “no”, your identity will remain anonymous but your review may still be made public. Do you want your identity to be public for this peer review? For information about this choice, including consent withdrawal, please see our [Privacy Policy](https://www.plos.org/privacy-policy). | Thank you for your option to publish the peer review history of this article. I opt to publish the peer review history of my article. Please proceed accordingly. |

- A marked-up copy of the manuscript highlighting the changes made in the original version and uploaded as a separate file named as ***Revised manuscript with Track changes.***
- An unmarked version without track changes has been furnished as ***Manuscript*** separately.
- I have completed the **Data Availability Statement** in the submission form
- I have finalized my **data sharing plan** and revised the statement accordingly.

I understand the importance of open data and have strived to comply with your policy.

Thank you again for your support of Open Science and for considering my work for publication in PLOS ONE.

Sincerely,

Safir Ullah

Corresponding Author
